# 

**Published:** 1897-07-31

**Authors:** 


NO ALKALIES USED.

### THE HOSPITAL

GLASGOW WESTERN INFIRMARY

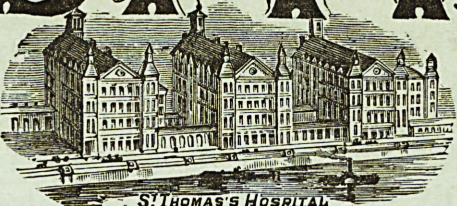

ST THOMAS'S HOSPITAL

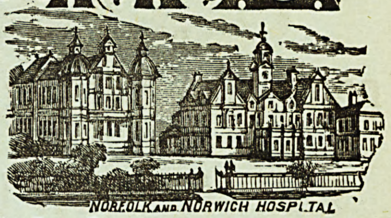

NORFOLK AND NORWICH HOSPITAL

No. 566. Vol. XXII. [Registered as a Newspaper.] SATURDAY, JULY 31ST, 1897. [Subscription Terms, see p. 298.] PRICE TWOPENCE.

